# Survey of chimeric IStron elements in bacterial genomes: multiple molecular symbioses between group I intron ribozymes and DNA transposons

**DOI:** 10.1093/nar/gku939

**Published:** 2014-10-16

**Authors:** Nicolas J. Tourasse, Fredrik B. Stabell, Anne-Brit Kolstø

**Affiliations:** 1 Laboratory for Microbial Dynamics (LaMDa), Department of Pharmaceutical Biosciences, University of Oslo, Oslo, Norway; 2 Institut de Biologie Physico-Chimique, UMR CNRS 7141, Université Pierre et Marie Curie, Paris, France

## Abstract

IStrons are chimeric genetic elements composed of a group I intron associated with an insertion sequence (IS). The group I intron is a catalytic RNA providing the IStron with self-splicing ability, which renders IStron insertions harmless to the host genome. The IS element is a DNA transposon conferring mobility, and thus allowing the IStron to spread in genomes. IStrons are therefore a striking example of a molecular symbiosis between unrelated genetic elements endowed with different functions. In this study, we have conducted the first comprehensive survey of IStrons in sequenced genomes that provides insights into the distribution, diversity, origin and evolution of IStrons. We show that IStrons have a restricted phylogenetic distribution limited to two bacterial phyla, the Firmicutes and the Fusobacteria. Nevertheless, diverse IStrons representing two major groups targeting different insertion site motifs were identified. This taken with the finding that while the intron components of all IStrons belong to the same structural class, they are fused to different IS families, indicates that multiple intron–IS symbioses have occurred during evolution. In addition, introns and IS elements related to those that were at the origin of IStrons were also identified.

## INTRODUCTION

Over the past decades it has been well recognized that mobile genetic elements have played a major role in genomic evolution. The spread of mobile elements has generated genomic and genic diversity that led to the creation of new combination of genes and new functions that have allowed organisms to survive or adapt to specific environments or conditions (see, e.g. (1–5)).

Insertion sequences (ISs in prokaryotes; class II DNA transposons in eukaryotes) are among the best known and widespread mobile elements. They are relatively simple and compact transposable DNA elements. A typical IS transposon is a piece of sequence ranging from 700 to 2500 bp (in prokaryotes) or >5000 bp (in eukaryotes) in length, encoding one or two open reading frames (ORFs), surrounded by 5′ and 3′ flanking sequences (often ending with terminal inverted repeats (IRs); for reviews see (6–11)). The ORFs encode transposases or other DNA recombinases such as tyrosine or serine recombinases or resolvases that are responsible for IS mobility within and between genomes, and they are usually highly specific to their cognate transposons. A few transposition mechanisms have been characterized, while the mechanisms of many IS elements are yet to be deciphered (for reviews see (6,8,11–15)).

IS elements are very diverse: more than 4000 prokaryotic ISs are currently known, divided into 26 families based on sequence similarity, organization, type of ends and type of ORF they encode (11,16–17). Similarly, thousands of DNA transposons, divided into 20 superfamilies, have been identified in eukaryotes (10,18). IS transposons differ greatly in the specificity of target site selection (19). Some IS elements are highly site-specific and insert at defined sequences, others have less stringent sequence requirements, while some ISs recognize structural motifs rather than sequence motifs. IS elements are present in virtually all eukaryotes and in most species of Bacteria and Archaea, and are particularly abundant in bacterial and archaeal plasmids. The distribution of IS transposons in genomes is ubiquitous, but the observed insertions are usually intergenic. This could be because some IS elements prefer intergenic regions or because intragenic insertions are selected out as they may be mostly deleterious. The number, type and location of IS vary greatly between different species, even between highly closely related ones.

**Figure 1.**
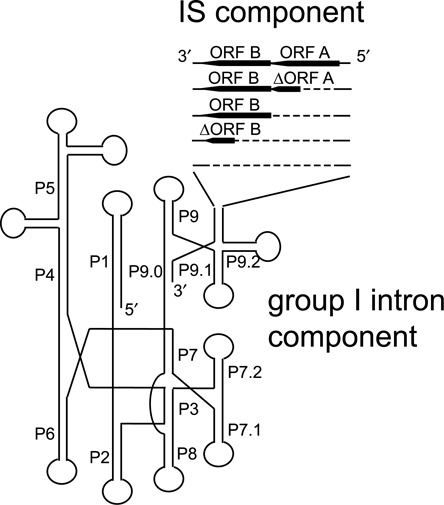
Schematic representation of the IStron structure. An IStron is made of two components: a group I intron and an IS element. The intron component is a self-splicing ribozyme made up of nine paired regions (P1–P9) which may include additional subdomains (P7.1, P7.2, P9.0, P9.1 and P9.2). The IS component is DNA transposon which encodes two ORFs (A and B; thick black arrowheads), one or both of them may be a transposase. In the majority of cases, variants have been identified, in which ORF A or both ORFs are truncated or missing.

Group I introns are self-splicing catalytic RNAs (ribozymes) that interrupt genes. They are able to catalyze their excision out of RNA transcripts and ligate their flanking RNA sequences (hereafter referred as exons). Group I introns are also mobile elements as they can reverse-splice into RNA and some contain an ORF encoding a homing endonuclease (HEG) that allows them to invade genomic DNA sequences. Group I introns have been extensively studied and the molecular details of the self-splicing and mobility reactions are well characterized (for reviews, see (20–24)). Group I ribozymes are 200–500 bp in length and fold into a secondary structure made up of usually 9–10 paired elements (called P1–P10, see Figure 1; (25–28)). When present, the HEG ORF is generally located within a terminal loop at the periphery of the structure. Splicing can occur via alternative pathways that lead to the release of a linear or circular (full-length or shortened) intron. During the splicing and circularization processes the intron RNA makes specific base-pairing contacts with its flanking exons; in particular the last 4–6 nucleotides (nt) of the 5′ exon pair with a complementary motif (the internal guide sequence, IGS) near the beginning of the intron to form the P1 domain which includes a critical and highly conserved U–G wobble base-pair at the 5′ splice site.

In contrast to IS elements, group I introns show remarkably specific distributions in genomes. Over 20 000 group I introns have been identified in Bacteria, bacteriophages and eukaryotes (organellar and nuclear genomes). In bacterial and eukaryotic chromosomes they are located in non-coding tRNA or rRNA genes, whereas in bacteriophages they are preferentially inserted in essential protein-coding genes, notably those involved in DNA metabolism or coding for phage structural proteins (22–23,29–32). In eukaryotic organelles, group I introns are found both in tRNA/rRNA and essential protein-coding genes from the photosynthesis or respiratory pathways. The locations of homologous introns are usually conserved among related species or strains. This is because HEGs are generally very site-specific as they recognize long target sites (14–40 bp; (33,34)), thus they promote intron insertions into cognate sites; this process is called homing. Another feature of group I introns is that they exhibit a great diversity in structure, and they are currently divided into 14 subclasses (25,31). In addition to HEG, group I introns can accommodate other sequence elements in their structure, as many introns are known to incorporate repeats, protein-coding and RNA genes, or even other ribozymes, which can bring the total intron length up to ∼20 kb (35–38).

In 2000, a research group identified a chimeric genetic element inserted within the enterotoxin-encoding *tcdA* gene of the bacterium *Clostridium difficile* (39). This element consists of a group I intron (without HEG) of subclass IA2 in its 5′ end fused in its 3′ end to a transposase-encoding IS element of the IS*200*/IS*605* family and was thus called an IStron (Figure 1). This particular IStron was named Cd*ISt*1 and was subsequently found in multiple copies in the chromosome of various *C. difficile* strains (39–41). A few years later our group identified a related element in chromosomes of bacteria belonging to the *Bacillus cereus* group, Bc*ISt*1 (42). Interestingly, for both Cd*ISt*1 and Bc*ISt*1 the localizations of the IStron copies are highly variable among strains and insertions are mostly intragenic and within unrelated protein-coding genes. Although IStron mobility has not been experimentally demonstrated, this variable distribution in bacterial chromosomes, which is unlike that of group I introns which are restricted to conserved tRNA and rRNA targets, strongly suggests that the IS element is responsible for IStron mobility. On the other hand, self-splicing of Cd*ISt*1 as a single unit containing the intron and IS (transposase) components has been confirmed (39,40). An IStron was therefore interpreted as a molecular symbiosis between unrelated genetic elements providing functions that are beneficial to both partners. The IS transposon confers mobility and allows the IStron to spread within and among genomes, while the splicing ability of the intron renders intragenic IStron insertions harmless.

Since the discoveries of Cd*ISt*1 and Bc*ISt*1, no further studies on IStrons have been reported and a number of questions regarding IStrons remain open. How abundant are they in genomes, how diverse are they, how did they originate and evolve? In addition, the mobility mechanism is unknown. To get insights into these issues, we have undertaken a comprehensive bioinformatic survey of IStrons in the large amount of available sequenced genomes, combining sequence comparisons with phylogenetic and structural analyses. We also conducted the first experimental study of IStron mobility, using Bc*ISt*1.

## MATERIALS AND METHODS

### Identification of IStrons

**Figure 2.**
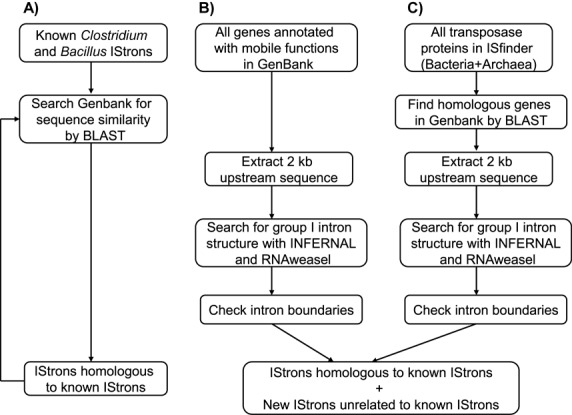
The 3-fold bioinformatic search procedure for IStron identification employed in this study (for details see the Materials and Methods section).

We searched for IStrons in public sequence databases using a 3-fold strategy involving searches at both the sequence and structural levels (Figure 2):

1. The first, basic, approach employed was to search the GenBank database (43) for elements showing nucleotide sequence similarity to the two known IStrons Cd*ISt*1 and Bc*ISt*1 using BLASTN ((44); Figure 2A). The nonredundant (nt) and whole genome shotgun sections of GenBank were searched and BLASTN (version 2.2.25) was run with default settings except for the following parameters: no filtering of low complexity regions (blastall option -F = F), a nucleotide match reward of 2 (-r = 2), an *e*-value of 10^−5^ (-e = 10^−5^) and the number of alignments to show set to a large value in order to keep all alignments (-v 1000000 and -b 1000000). This approach mainly identified IStron copies that were closely related to Cd*ISt*1 and Bc*ISt*1 in both the group I intron and IS element components. In addition, when there was a significant hit to the intron part only (of 100 nt or more), the subject sequence was retrieved along with downstream sequence (extending to two or three genes downstream of the intron hit) to check whether it could represent an IStron with an intron component related to that of Cd*ISt*1 and Bc*ISt*1 but associated with a different IS component. All IStrons identified by the above procedure were then used as queries to search for additional relatives the same way.
2. Second, in order to identify IStrons that may not be closely related in sequence to Cd*ISt*1 and Bc*ISt*1 a more generic approach was designed (Figure 2B). It consisted in searching for group I introns that are located next to mobile genes. For this, all genes in GenBank (nr and env_nr sections) coding for proteins annotated with functions related to DNA mobility were compiled, based on the following annotation keywords: IS element, excision, excisionase, insertase, insertion, integrase, integrate, integrative, integration, inversion, invertase, mobile, recombinase, recombination, resolution, resolvase, transposable, transposase, transposition, transposon (HEG was not included since HEGs are commonly found as part of typical group I introns). For each of these genes, the nucleotide sequence was retrieved along with 2 kb of upstream sequence. Then, the INFERNAL (45) and RNAweasel/ERPIN systems (35,46) were used to search for a group I intron ribozyme structure within the sequence upstream of the putative mobile gene. INFERNAL and ERPIN apply the two most sensitive algorithms designed to identify structured RNAs not on the basis of primary sequence but based on patterns in the secondary structure. INFERNAL and ERPIN use covariance models (CMs) and structure profiles, respectively, which are computed from curated RNA sequence alignments. For INFERNAL (version 1.0.2), introns were searched for using the cmsearch program run with default settings and the group I intron CM available in the Rfam database (RF00028; http://rfam.sanger.ac.uk/; (47)). Hits were considered significant when the score was 20 or higher (the associated *e*-value and *P*-value were < 10^−7^ and < 10^−9^, respectively). For ERPIN, searches were conducted on-line using the RNAweasel web service (http://megasun.bch.umontreal.ca/RNAweasel/; (35)). When a group I intron structure was identified upstream of a given mobile gene, the sequence of the whole locus was manually examined to determine whether it was an actual IStron or whether it was simply an intron located next to a mobile element. The whole search procedure detailed above should identify any type of IStrons, including the known Cd*ISt*1 and Bc*ISt*1 elements and their close relatives that were identified in (1). The latter IStrons thus served as ‘positive controls’ to validate the generic procedure.
3. The third approach employed to identify IStrons was basically the same as in (2) but using the ISfinder database (http://www-is.biotoul.fr/; (17,48); Figure 2C) to collect additional mobile genes. ISfinder provides a curated and annotated set of several thousands IS elements from Bacteria and Archaea, and therefore likely includes IS transposases/recombinases that have been missed by the annotation keyword search performed in (2). Thus, the protein sequences of the ORFs encoded by all IS elements listed in ISfinder were searched against GenBank (nr and env_nr sections) to retrieve these proteins and their homologues (using BLASTP version 2.2.25 with default settings except no filtering of low complexity regions, an *e*-value of 10^−5^, and the number of alignments to keep set to 1000000, and proteins were considered homologous if the BLASTP hit covered 50% or more of the length of the query or subject sequence). The corresponding nucleotide sequences were extracted along with 2 kb of upstream sequence. Then, group I intron structures and IStrons were identified and evaluated as described in (2) above.

Programmatic search and retrieval of GenBank sequences was performed using the EPost, ESearch and EFetch functions of the NCBI Entrez programming utilities (E-utilities; http://eutils.ncbi.nlm.nih.gov/entrez/eutils/). In all analyses described above manual examination was necessary to confirm that a given group I intron–IS pair was an actual IStron. A critical point was to find out whether the 3′ end of the intron was located upstream or downstream of the mobile gene (which would correspond respectively to independent elements or a putative IStron). For that, group I intron and IStron boundaries were determined in three ways: (i) by similarity with the boundaries of known introns and IStrons; (ii) by aligning the regions flanking the intron–IS sequence with homologous sequences from other strains or species not containing the intron–IS segment at that locus; (iii) or by folding the intron secondary structure (see below; in the case of an IStron, the IS component would be entirely included within the intron structure).

Novel IStrons were named according to the nomenclature proposed by Hasselmayer *et al.* (40), where the first two letters are initials of the genus and species, followed by the keyword *ISt*, followed by a number, e.g. Cd*ISt*1 for *C. difficile* IStron 1.

### Secondary structure analysis

The secondary structure of group I intron RNAs (ORFs removed) from IStrons was computationally predicted by constrained folding using the MFOLD version 3.1.2 package (49) following the structures of introns from the IA2 class (25,31). That is, conserved and identifiable sequence motifs corresponding to the structure of group IA2 introns were forced during the folding computation.

### Phylogenetic analysis of IStron ORFs

The ORFs from the IS components of IStrons were searched against the ISfinder database (17,48) using BLASTP or BLASTX to determine the IS families they may belong to, based on the family of their closest relative in ISfinder. BLASTP and BLASTX were run with default parameters with the exceptions of no filtering of low complexity regions. For each identified IS family, a multiple protein sequence alignment of IStron ORFs and their relatives from IS elements was computed using CLUSTALW 2.0 (50) followed by manual correction. Nonhomologous or ambiguously aligned N- and C-terminal regions were removed. A phylogenetic tree was reconstructed by means of the maximum-likelihood method using the best-fit amino-acid substitution model. Model estimation and tree reconstruction were carried out using the ReplacementMatrix webserver (51). Statistical confidence in branchings was computed as Shimodaira–Hasegawa (SH)-like support values, which represent one type of approximate likelihood ratio test (52,53).

### DNA and RNA isolation

Isolation of *B. cereus* ATCC 10987 RNA was performed as described in (54), except that samples were taken out after 3, 4.5 and 6 h. These time points represent the early phase, and the mid- and late-exponential phase of the growth curve, respectively.

Genomic DNA of *B. cereus* ATCC 10987 was isolated as in (55).

*Escherichia* *coli* plasmid DNA was isolated using Qiaprep Spin Miniprep Kit (Qiagen) as described by the supplier.

### PCR and RT-PCR

PCR and RT-PCR were carried out as described in (55) with primers listed in Supplementary Table S2. For TA-cloning Dynazyme was added in an extra extension step for the addition of A-overhangs to PCR products.

### Cloning and mutagenesis

RT-PCR or PCR products were gel-purified from 1X TAE gel (QIAquick gel extraction Kit, Qiagen), and were cloned into pCR2.1-TOPO TA-cloning vector (Invitrogen) and subsequently sequenced. The vector contains an IPTG-inducible P*lac* promoter to drive expression of the insert. It also includes a single *Nco*I site that was used for linearizing the plasmid, and two *Eco*RI sites used for plasmid digestion (see Figure 7A).

**Figure 3.**
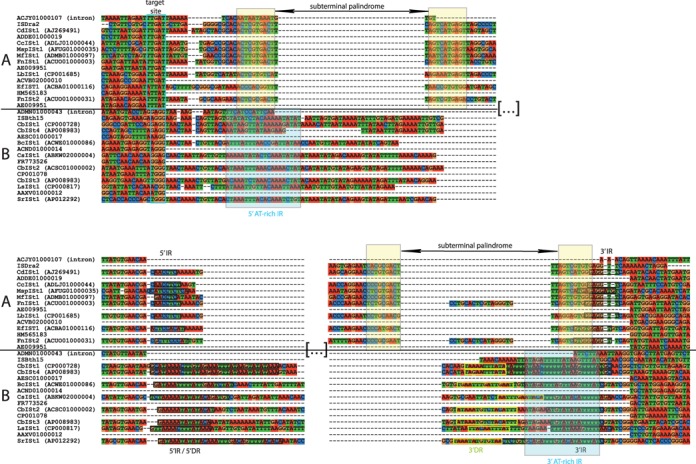
Multiple sequence alignment showing the IStron target sites, boundaries and sequence ends. Omitted regions are indicated by the sign ‘[…]’. GenBank accession numbers of genomic sequences encoding the IStrons shown are given in parentheses next to IStron names. IStron-less sequences homologous to IStron flanking exons are included to confirm IStron boundaries (sequences named by their GenBank accession numbers only). Also included are IS elements (IS*Dra2* and IS*Bth15* from *D. radiodurans* and *B. thuringiensis*, respectively, taken from the ISfinder database) and group I introns (GenBank accessions ACJY01000107 and ADMN01000043 from *F. periodonticum* and *T. sanguinis*, respectively) with ends and target sites similar to those of IStrons. Group A and B IStrons are inserted next to T-rich pentanucleotide and GG-containing sites, respectively, which are boxed in cyan. Note that the target site of group B IStrons does not end with a U, unlike for virtually all known group I introns. In group A IStrons, the regions corresponding to the subterminal palindromes that are recognized by the ORF A transposase during transposition of IS*200*/IS*605* elements are highlighted in yellow. For group B IStrons, imperfect AT-rich IR motifs that are presumed to be recognized by the ORF A transposase during transposition of IS*607* elements are highlighted in light blue. IR motifs forming the IR stem in the group I intron structure of IStrons (see Figure 4) are boxed in black. The direct repeat sequence (3′ DR) that is similar to part of the 5′ IR motif (5′ IR/5′ DR) and that is located immediately upstream of, and is globally complementary to, the 3′ IR motif in group B IStrons is boxed in yellow.

**Figure 4.**
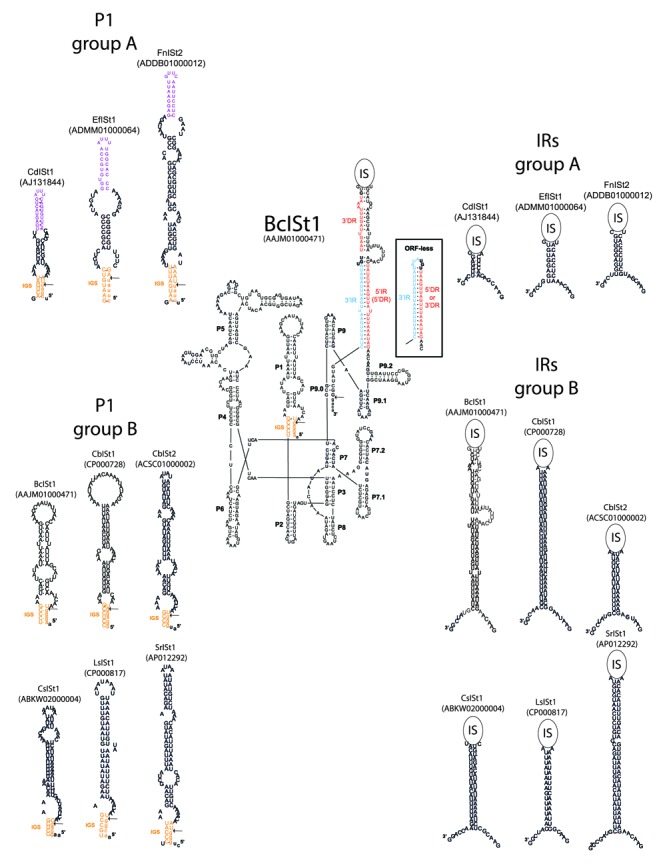
Predicted secondary structure of the Bc*ISt*1 IStron and comparison of key structural features of group A and B IStrons. In the Bc*ISt*1 structure, labels P1 to P9 indicate the group I intron domains and subdomains. The IR and DR motifs in the IR stem-loop region of Bc*ISt*1 are colored in red and blue. The boxed inset shows the predicted structure of IRs in the ORF-less copies of Bc*ISt*1 that were obtained experimentally in this study (see Figure 7) and that were also identified in the genome sequences of emetic strains of *B. cereus*. The IGS stem is colored in orange. Exon sequences are in lowercase. Splice sites are indicated by arrows. A comparison of the P1 subdomains of various IStrons is shown to illustrate that the U–G pair that is critical for 5′ splice site recognition is surrounded by flanking base-pairs within the IGS stem in group A IStrons, whereas it is predicted to be immediately followed by an internal loop in group B elements, where it is shifted by one bp relative to the splice site. For group A IStrons, bases corresponding to the subterminal palindromes that are required for IS*200*/IS*605* elements (see Figure 3) are highlighted in purple. A comparison of the IR regions is also shown to illustrate that the IR stem is short and compositionally balanced in group A IStrons, in contrast to the long and extremely AU-rich stem in group B elements. The IS (transposase) component is represented by a circle. The structural models shown are from selected representatives that illustrate the variability in sequence and structure. Sequences of the 5′ and 3′ ends of the other IStrons, which are highly similar to those shown here, can be seen in Figure 3. GenBank accession numbers of genomic sequences encoding the IStrons shown are given in parentheses.

**Figure 5.**
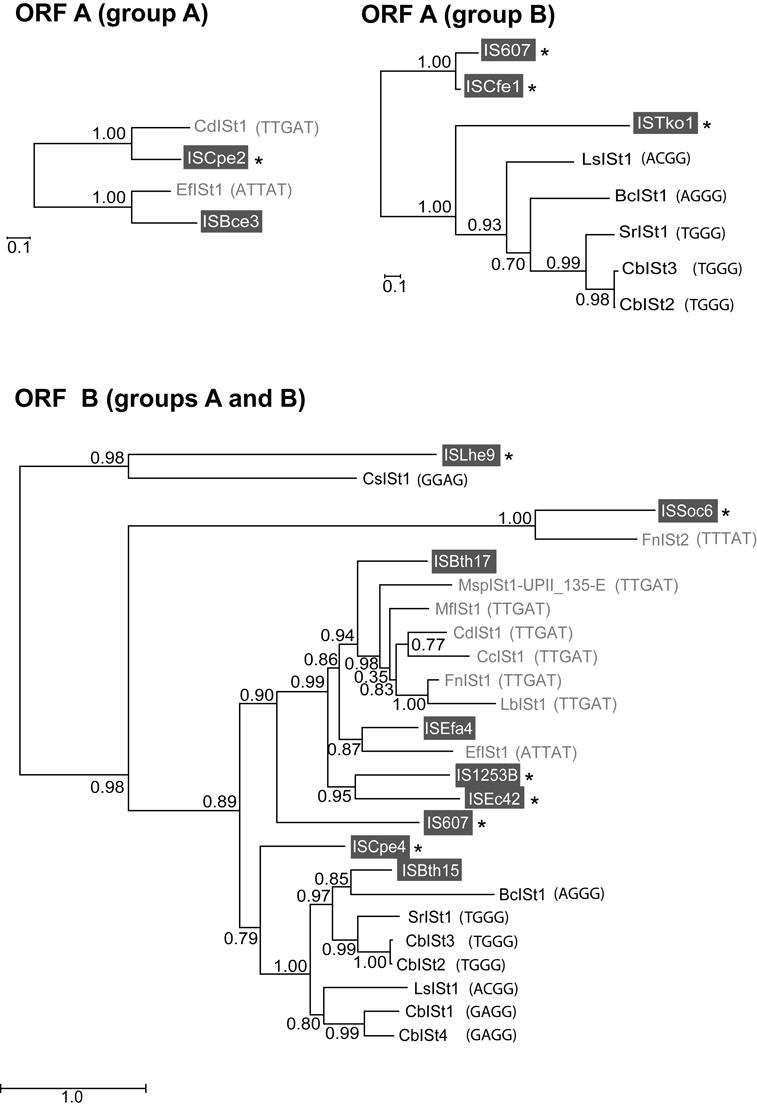
Phylogenetic analysis of ORFs A and B from IStrons and IS elements. Trees were reconstructed using the maximum-likelihood method. Only IS ORFs that are the closest relatives to IStron ORFs are included (listed in Table 1). IStrons from groups A and B are labeled in gray and black, respectively, and their target sites are given in parentheses. IS elements are labeled in white over a gray background. IS elements marked with an asterisk are from species in which no IStron could be identified. As can be seen, IStron ORFs usually do not form monophyletic clusters separated from IS elements and IStrons from groups A and B are intermixed, indicating that IStrons have multiple origins. Note also the correlation between IStron ORF phylogeny and target site. Numbers on nodes represent statistical confidence in branchings computed as SH-like support values. Scale bars are in average numbers of amino-acid substitutions per site. The number of amino-acid positions used in the analysis was 135, 206 and 441 for the ORF A (group A), ORF A (group B) and ORF B datasets, respectively.

**Figure 6.**
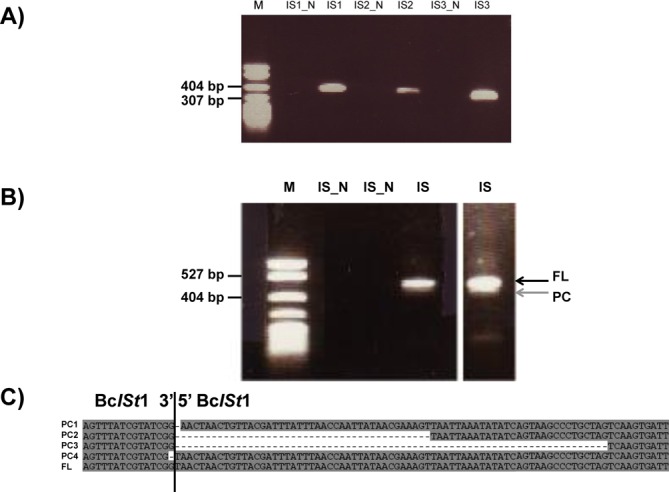
Bc*ISt*1 IStron splicing *in vivo* in *B. cereus* ATCC 10987. (**A**) RT-PCR experiments conducted on total RNA with inward primers located in the exons flanking the IStron. For each of the three Bc*ISt*1 copies encoded by *B. cereus* ATCC 10987 (lanes IS1, IS2 and IS3), a product of ∼300–400 bp corresponding to the ligated exons was detected, indicating that the 1.9-kb IStron has spliced out (expected product sizes: 382, 356 and 323 bp for IS1, IS2 and IS3, respectively). Lanes IS1_N, IS2_N and IS3_N represent negative control experiments conducted without RT. Lane M, size marker. (**B**) RT-PCR experiments conducted with outward primers located in the IStron. Products of ∼400 bp were detected, corresponding to the junction of excised, circular IStron forms. Black and gray arrows indicate junctions corresponding to the full-length (or near full-length, FL) and partial (PC) IStron circles, respectively. IS_N, negative control without RT; M, size marker. DNA marker in A and B: pBR322-*Msp*I (New England Biolabs). (**C**) Sequences of circular junction products include the full-length IStron circle (FL, bottom sequence) as well as several partial circles (PC) lacking the first one, the first 42, the first 71 or the last base.

**Figure 7.**
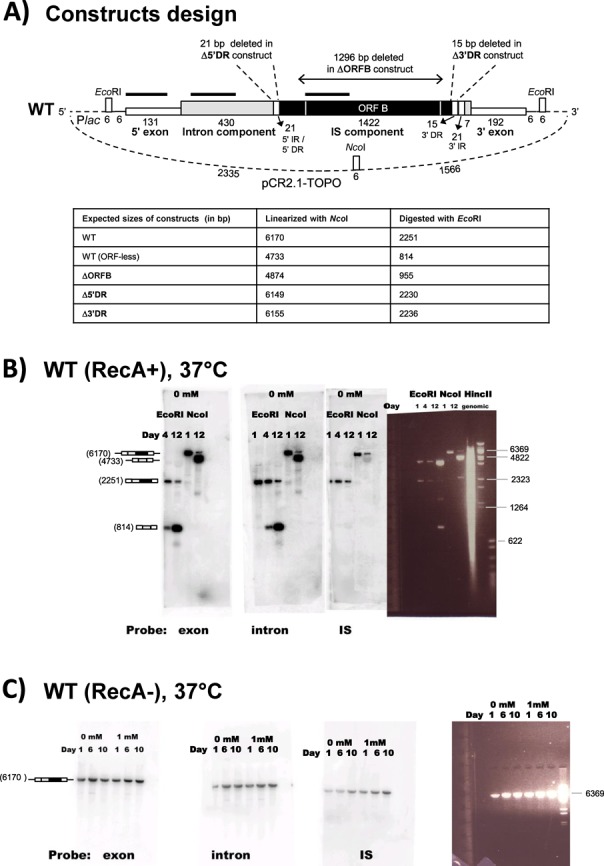
(**A**) Constructs made to test the mobility of the Bc*ISt*1 IStron. Constructs were cloned into pCR2.1-TOPO TA-cloning vector. The various sequence features are indicated, along with their sizes (in bp) and the locations of restriction sites used for plasmid linearization or digestion. The size indicated for the IS component does not include the DR and IR motifs since it is not known if these motifs belong to the intron or the IS component. The thick horizontal black lines indicate the positions of hybridization probes. P*lac* is an IPTG-inducible promoter. Features are not drawn to scale. The expected sizes of all constructs are listed in the table underneath. (**B** and **C**) Test for Bc*ISt*1 IStron mobility using the WT Bc*ISt*1 construct at 37°C in a RecA+ (xl–1; panel B) or RecA− (SCS110; panel C) *E. coli* strain. The various gels show the results of Southern hybridization experiments conducted with labeled dsDNA probes specific for the intron or IS (ORF B) component of Bc*ISt*1 or the 5′ exon. Schematic structures of the products in relevant bands are drawn on the left and their expected sizes (in bp) are given in parentheses; white boxes: exons, gray box: intron component, black box: IS component, black horizontal line: vector. The rightmost panel shows ethidium bromide straining of plasmid DNA, with the sizes (in bp) of relevant bands indicated. Plasmid DNA was linearized with *Nco*I (panels B and C) or digested with *Eco*RI (panel B). Hybridizations were carried out on plasmid DNA samples extracted from bacterial cultures during 8–12 days of bacterial growth in LBamp media without IPTG or with 1 mM IPTG. Panel B also shows hybridization of genomic DNA isolated at the end of the growth assay and purified of plasmid. Genomic DNA was digested with *Hinc*II. A difference among the banding patterns obtained with the various probes would indicate that a DNA rearrangement (i.e. an excision event) involving the IStron has occurred. In panel B, the loss of hybridization signal with the IS ORF B probe for the lower band (corresponding to the ORF-less product) implies excision of part of the IS component. DNA markers: Lambda-*BstE*II and pBR322-*Msp*I (New England Biolabs).

Wild-type (WT) construct was made with primer pair BcISt1c left/right for amplification before cloning. The deletion constructs Δ5′DR, Δ3′DR and ΔORFB were amplified by inverse PCR with outward primers (see Supplementary Table S2) from the WT plasmid construct as template using Pfu Turbo in order to remove specific IStron regions, and then ligated with T4 ligase (New England Biolabs). The various constructs were transformed into either *E. coli* xl-1 (recA+) or *E. coli* SCS110 (recA−) strains.

### Growth of transformed *E. coli*

A single colony of cells transformed with a given plasmid construct were inoculated in LBamp (100 μg/ml ampicillin or 59 μg/ml kanamycin) overnight, with a following inoculation to give an optical density of 0.05 at 600 nm in fresh LBamp, pH 7, with or without 1 mM IPTG, 230 rpm, at either 30, 37 or 42°C. After the culture had grown for ∼4 h part of the culture was stored in glycerol (10%) at −20°C, while the rest was used to isolate plasmids. The frozen culture was used to inoculate overnight an LBamp culture (without IPTG). Inoculation, isolation of plasmid and freezing with the same culture was continued for 8–12 days. The whole assay was repeated three times.

### Southern blotting and hybridization

PCR products (made with primers listed in Supplementary Table S2) used as probes in Southern hybridizations were agarose gel-purified using the QIAquick Gel Purification kit (Qiagen). The probes were labeled with α^32^P-dCTP (Amersham) using NetBlot Kit (New England Biolabs).

Genomic DNA (∼1–10 μg) was digested to completion with *Hinc*II and plasmid DNA (∼1–2 μg) was digested to completion with *Eco*RI or *NcoI* and run on a 0.8% agarose gel. After electrophoresis, DNA was transferred to nylon membrane (Hybond N+, Amersham) by capillary blotting overnight. Hybridization was performed with Perfecthyb Plus (Sigma) as described by the supplier, with the highest stringency wash. Membranes were exposed overnight, and signals were visualized using a phosphorimager (STORM 860, Molecular Dynamics).

## RESULTS

### Identification of novel IStrons in bacterial genomes

The upstream regions of 584 685 genes annotated with functions involved in mobility processes (such as transposase, recombinase, resolvase, integrase, invertase or excisionase), and the homologues of these genes, were screened by bioinformatic tools for the presence of a group I intron, which, together with the mobile gene, could form a composite IStron element (Figure 2). Altogether, 294 full-length IStrons (and 115 partial copies) were found in Bacteria and bacteriophages, whereas none could be identified in Archaea and eukaryotes. Remarkably, in Bacteria IStrons were present in 117 strains from 18 species that belong to only two different phyla, the Firmicutes (low G+C Gram-positive bacteria) and the Fusobacteria (obligate anaerobe Gram-negative; Table 1). Most of the IStrons were copies of the two previously known Cd*ISt*1 and Bc*ISt*1 elements (170 and 69 copies, respectively) from multiple strains of *C. difficile* and the *B. cereus* group, respectively (39,42). The other 55 full-length IStrons were copies representing 14 novel, different, IStron elements. All IStrons identified were composed of a group I intron associated with an IS element; no other type of mobile element was found as part of IStrons.

**Table 1.** Identified full-length IStrons^a^

IStron	Species (phylogenetic phylum, class)	Size (bp)^b^	No. of copies per strain^c^	Group I Intron class	IS element ORF composition^d^	IS element family	Closest IS ORF relative (% aa identity)^e^
Group A							
Cd*ISt*1	*Clostridium difficile* (Firmicutes, Clostridia)	2213	9–24	IA2	ORF A + ORF B, ΔORF A + ORF B, ORF B only, ΔORF B only	IS*200*/IS*605*	ORF A: IS*Cpe2* (66%)
							ORF B: IS*Bth17* (52%)
Cc*ISt*1	*Clostridium citroniae* (Firmicutes, Clostridia)	1779	1	IA2	ΔORF A + ORF B	IS*200*/IS*605*	ORF B: IS*Bth17* (47%)
Ef*ISt*1	*Enterococcus faecium* (Firmicutes, Bacilli)	2140	1	IA2	ORF A + ORF B	IS*200*/IS*605*	ORF A: IS*Bce3* (64%)
							ORF B: IS*Efa4* (49%) / IS*Ec42* (39%)
Msp*ISt*1-UPII 135-E	*Megasphaera sp.* UPII 135-E (Firmicutes, Negativicutes)	1802	1	IA2	ΔORF A + ORF B	IS*200*/IS*605*	ORF B: IS*Bth17* (46%)
Mf*ISt*1	*Megamonas funiformis* (Firmicutes, Negativicutes)	1839	1	IA2	ΔORF A + ORF B	IS*200*/IS*605*	ORF B: IS*Bth17* (52%)
Fn*ISt*1	*Fusobacterium nucleatum subsp. polymorphum*, *Fusobacterium nucleatum subsp. animalis* (Fusobacteria, Fusobacteriia)	1857	3–5	IA2	ΔORF A + ORF B	IS*200*/IS*605*	ORF B: IS*Bth17* (50%)
Fn*ISt*2	*Fusobacterium nucleatum subsp. polymorphum*, *Fusobacterium nucleatum subsp. animalis* (Fusobacteria, Fusobacteriia)	2045	1–6	IA2	ΔORF A + ORF B	IS*200*/IS*605*	ORF B: IS*Soc6* (37%)
Lb*ISt*1	*Leptotrichia buccalis* (Fusobacteria, Fusobacteriia)	1760	1	IA2	ΔORF A + ORF B	IS*200*/IS*605*	ORF B: IS*Bth17* (50%)
Group B							
Bc*ISt*1	*Bacillus cereus* group (*B. cereus*, *B. thuringiensis*, *B. anthracis*, *B. weihenstephanensis*, *B. mycoides*, *Bacillus sp.* 7_6_55CFAA_CT2; Firmicutes, Bacilli)^f^	2391	1–3	IA2	ΦORF A + ORF B, ΔORF A + ORF B, no ORF	IS*607*	ORF A: IS*Tko1* (41%)
							ORF B: IS*Bth15* (40%) / IS*1253B* (30%)
Ls*ISt*1	*Lysinibacillus sphaericus* (Firmicutes, Bacilli)	2438	1	IA2	ORF A + ORF B	IS*607*	ORF A: IS*Tko1* (37%)
							ORF B: IS*Bth15* (45%)
Sr*ISt*1	*Selenomonas ruminantium subsp. lactilytica* (Firmicutes, Negativicutes)	2583	4	IA2	ORF A + ORF B	IS*607*	ORF A: IS*Cfe1* (44%)
							ORF B: IS*Bth15* (49%)
Cb*ISt*1	*Clostridium botulinum* (Firmicutes, Clostridia)	1378	1	IA2	ΔORF A + ORF B	IS*607*	ORF B: IS*607* (40%)
Cb*ISt*2	*Clostridium botulinum* (Firmicutes, Clostridia)	2055	1	IA2	ORF A + ORF B	IS*607*	ORF A: IS*Cfe1* (44%)
							ORF B: IS*Bth15* (54%)
Cb*ISt*3^g^	*Clostridium botulinum* phage c-st	2559	1	IA2	ORF A + ORF B	IS*607*	ORF A: IS*Cfe1* (44%)
							ORF B: IS*Bth15* (53%)
Cb*ISt*4	*Clostridium botulinum* phage c-st, *Clostridium botulinum* phage D-1873	2053	1	IA2	ΔORF A + ORF B	IS*607*	ORF B: IS*Bth15* (51%)
Cs*ISt*1	*Clostridium sporogenes* (Firmicutes, Clostridia)	2369	1	IA2	ORF B only	IS*607*	ORF B: IS*Lhe9* (30%)

^a^A set of 115 partial IStron copies highly similar to full-length copies were also identified in strains of the species listed above, with the addition of *Fusobacterium periodonticum*. The partial IStrons consisted mostly of the group I intron or the IS component. These sequences were located at the extremities of genomic scaffolds and therefore likely represent unassembled pieces of additional full-length IStron copies.

^b^Size of the longest copy identified.

^c^GenBank accession numbers and genomic coordinates of all copies are given in Supplementary Table S1.

^d^ΔORF means that the ORF is truncated; ΦORF means that the ORF is full-length but frameshifted.

^e^Based on the best hit from a BLASTP or BLASTX search of the ISfinder database using the IStron ORFs as queries. Best hits are given only for full-length IStron ORFs. When the best hit is from the same species as that harboring the IStron, the best hit to an IS from a different species is also given after a ‘/’ character.

^f^Species of the *B. cereus* group are very closely related at the genomic level and are phylogenetically intermixed (58,59), and are therefore treated here as a single group.

^g^Cb*ISt*3 is reported as an IS element IS*Cbt4* in ISfinder.

The IStron copy number per strain is usually small, ranging from 1 to 6, except in strains of *C. difficile* which can contain up to 24 IStrons (Table 1). Strains of a given species usually harbor highly similar copies (generally >93% nt sequence identity over the entire length) of only one particular IStron, but there are exceptions such as *Clostridium botulinum*, *Fusobacterium nucleatum subsp. polymorphum* and *F. nucleatum subsp. animalis*, in which two different IStron elements coexist. With respect to replicons, 154 of the 294 full-length IStron copies identified were located on bacterial chromosomes, whereas only three copies were found on plasmids (Cb*ISt*1, Cb*ISt*2 and Sr*ISt*1; see Supplementary Table S1). Two IStrons, Cb*ISt*3 and Cb*ISt*4, were located in *C. botulinum* bacteriophages (Supplementary Table S1; Cb*ISt*3 is reported as IS*Cbt4* IS element in ISfinder). The remaining 134 IStron copies were in unfinished and/or unclassified genomic scaffolds, most of them are likely to be chromosomal based on sequence similarity with related genomes. It has been previously observed that the genomic distributions of Cd*ISt*1 and Bc*ISt*1 in *C. difficile* and *B. cereus* group species, respectively, were highly variable among species and strains, and that most insertions were intragenic and within genes coding for unrelated functions located throughout the genome (39,42). The distribution of the newly identified IStrons confirms that this is a general pattern. Out of 202 insertion sites for which gene predictions were available, 118 (i.e. 58%) were intragenic, and the target genes encoded diverse functions, such as drug or metal transporter, alcohol dehydrogenase, quinone oxidoreductase, DNA helicase, transcriptional regulator or hypothetical protein (Table 1 and Supplementary Table S1). Moreover, 64 (i.e. 54%) of the intragenic insertions were in-frame with the host gene (Supplementary Table S1).

The group I intron components of all IStrons share a few conserved sequence motifs but are quite similar mainly at the structural level. Their secondary structures show several features of the IA2 class (25,31), in particular the presence of two hairpins in domain 7 (P7.1 and P7.2) and an extended domain 9 with three paired regions (P9.0, P9.1 and P9.2), as described previously for Cd*ISt*1 ((39), Figure 4). This indicates that the intron components of all IStrons are derived from a common ancestral ribozyme. Furthermore, all IStrons have a similar organization. In all elements the IS component is located at the same relative position within the group I intron structure, i.e. downstream of the P9.2 subdomain after a GAACGA, GAACAA, GAACAC, GAAUAA or GAAUAU motif (Figure 3). The region encoding the ORFs is flanked by IR motifs that are predicted to form an AU-rich stem-loop structure (Figure 4).

The IS components of IStrons are not well conserved. Their coding capacity is variable, which makes the IStron size range from 482 to 2582 nt in length (Table 1 and Supplementary Table S1). A few IStron copies encode two full-length ORFs, hereafter referred to as ORF A and ORF B, both located on the forward strand, while in many copies ORF A is either frameshifted, truncated or missing (Table 1). In addition, a few Cd*ISt*1 copies harbor a truncated ORF B, while the most extreme case was observed in Bc*ISt*1 variants from emetic *B. cereus* strains which have lost both ORFs and part of the sequence flanking the ORFs and only retain the sequence forming the IR stem-loop structure (Figure 4, boxed inset). Interestingly, in IStrons with degenerated or missing ORFs A Cd*ISt*1, Fn*ISt*1, Fn*ISt*2 and Bc*ISt*1, the modified or deleted regions are identical among IStron copies and multiple copies of the same degenerated IStron are found in different genomic locations, which suggests that the particular modification or deletion occurred once in an ancestral copy and that the IStron has been able to spread without ORF A. In contrast, no IStron harbored a complete ORF A and a degenerated ORF B. The Bc*ISt*1 variants lacking full-length copies of both ORFs were identified in five *B. cereus* strains (AH187, H3081.97, NC7401, BDRD-ST26 and Q1; Supplementary Table S1) that are phylogenetically closely related as they are part of a cluster of emetic strains (56,57,58,59) and their IStron copies are located in orthologous loci. This indicates that the ORF-less IStrons have been transmitted vertically among these strains.

### Two major groups of IStrons

Based on the target site, the IStron boundaries and the IS family, the 16 different IStron elements could be divided into two major groups named A and B. Each group exhibited features similar to those of either Cd*ISt*1 or Bc*ISt*1. Group A includes Cd*ISt*1 and IStrons from *C. citroniae*, *Enterococcus*, *Megasphaera*, *Megamonas* and *Fusobacterium*, while Bc*ISt*1 belongs to group B along with IStrons from *Lysinibacillus*, *Selenomonas*, *Clostridium sporogenes*, *C. botulinum* and *C. botulinum* phages (Table 1). Note that no species harbors IStrons from both groups. Group A IStrons are inserted downstream of a T-rich pentanucleotide TTGAT, ATTAT or TTTAT, and their 3′ terminus ends with TCAG, as originally reported for Cd*ISt*1 ((39), Figure 3). Group B IStrons exhibit a different pattern. Like Bc*ISt*1 (42), they are inserted after GG-ending sites, such as AGGG, TGGG or GAGG, and terminate with CGG at the 3′ end. The Cs*ISt*1 IStron of *C. sporogenes* deviates slightly from the group B consensus, as it is inserted downstream of an AG site and ends with CAG. In contrast to the conservation of the 5′ flanking sequences, the nucleotides 3′ of the IStrons are highly variable, indicating that IStrons do not duplicate their target sequence upon insertion.

IStrons differ in the first 100 nt of their 5′ ends which span the P1 and P2 domains of the intron component. In group I introns P1 is involved in the recognition of the 5′ splice site via the formation of the IGS with the 5′ exon. It is remarkable that for elements of group B the last nucleotide of the 5′ exon is a G, while it is a U in group A elements and also virtually always a U (or exceptionally a C) in the thousands of known group I introns (30–32). There is a U at the 5′ exon-intron junction of group B IStrons, but comparisons of the flanking exons with homologous IStron-less sequences in other species or strains confirmed that the U base was not part of the 5′ exon and thus must be part of the IStron sequence (Figure 3). This was further confirmed by experimental evidence for Bc*ISt*1 (see below). Group B IStrons thus seem to exhibit an unusual 5′ splice site. In group I introns the U base at the 5′ exon-intron junction is part of the critical U–G pair that signals the 5′ splice site. Interestingly, there appears to be a correlation between the structure of the P1 domain and the IStron group. In all group A elements, the U–G pair is surrounded by flanking base-pairs within the IGS stem (the typical situation in group I introns), whereas in all group B elements (except Sr*ISt*1) it is predicted to be immediately followed by an internal loop (Figure 4). Another noticeable, though more subtle, difference between the two IStron groups is that the IR stem-loop structure at the 3′ end enclosing the ORF region is remarkably long and extremely AU-rich in group B IStrons (15–34 base-pairs, >80% AU pairs) compared to a much shorter and compositionally balanced structure in group A elements (6–10 base-pairs with equal numbers of CG and AU pairs; Figures 3 and 4). In addition, in group B IStrons a sequence that, depending on the IStron, is strongly or weakly similar to part of the 5′ side of the IR stem is directly repeated on the 3′ side immediately upstream of the 3′ IR motif (boxed in yellow in Figure 3). This sequence is globally complementary to the 3′ IR motif and could be predicted to form an RNA stem with the 3′ IR as in the case of the ORF-less Bc*ISt*1 IStrons (see Figure 4).

With respect to the IS component, it has been shown that ORF A and B of Cd*ISt*1 were respectively homologous to transposases of the IS*200* and IS*1341* subgroups of the IS*200*/IS*605* family of IS elements, and that several features of Cd*ISt*1 were also typical of the IS*200*/IS*605* family, including insertion downstream of a conserved T-rich pentanucleotide motif, a target specificity at the 5′ but not the 3′ end, no target site duplication and lack of terminal IRs (6,8,11,39,41,60). Here we show that this is true for all group A IStrons. A BLAST search of the ISfinder database (17,48) indicated that the closest relatives of ORFs A and B from group A IStrons were ORFs A and B from IS elements belonging to the IS*200*/IS*605* class such as IS*Bth17* and IS*Soc6* (Table 1). IS*200*/IS*605* ORFs A are tyrosine recombinases (61,62). In contrast, whereas the ORFs B of all group B IStrons were also related to ORF B proteins of the IS*200*/IS*605* family (e.g. ORF B of IS*Bth15*), their ORFs A were homologous to ORFs A of the IS*607* class (e.g. ORF A of IS*Cfe1*), and belong to the family of serine recombinases (63,64). It should be noted that IS elements of the IS*200*/IS*605* and IS*607* families are chimeric and are composed of two ORFs of different phylogenetic origins, and that the ORFs B of the two families exhibit sequence homology whereas the ORFs A are completely unrelated (6,60,65–66). That is, IS*607* elements encode a specific ORF A and an ORF B homologous to IS*200*/IS*605*. Therefore, the fact that group B IStrons encode a IS*607*-like ORF A and a IS*200*/IS*605*-like ORF B suggests that the IS component of these IStrons belongs to the IS*607* family. Interestingly, IS*607* transposons insert in GG-containing targets (65), as is the case for group B IStrons (Figure 3).

### Multiple origins of IStrons

An important question about IStrons relates to their origin. The fact that there are two groups of IStrons with different target sites and harboring IS elements of different families would suggest that the association between group I introns and IS elements occurred at least twice in evolution. In order to get insights into the origin and evolution of IStrons, we studied the phylogenetic relationships between IStrons and their closest group I intron and IS element relatives.

First, it should be noted that no separate copies of the intron and IS components of IStrons were found in any genome; all identified copies were always part of IStrons. However, group I introns and IS elements showing homology to those from IStrons but corresponding to different elements were identified. Braun *et al.* (39) previously showed that the IS element IS*8301*/IS*Dra2*, an IS*200*/IS*605* family member from *Deinococcus radiodurans*, in addition to encoding two ORFs with significant amino-acid sequence similarity (63–79%) to those of Cd*ISt*1, a group A IStron, shared the same target site (TTGAT) and an homologous 5′ end with the IStron. We noticed here that the 3′ ends of IS*8301*/IS*Dra2* and Cd*ISt*1 are also similar (Figure 3). A characteristic of IS*200*/IS*605* family transposons is that both ends contain subterminal palindromes that are recognized by ORF A to mediate single-stranded DNA transposition (67–69), and, interestingly, the regions homologous between both ends of IS*8301*/IS*Dra2* and Cd*ISt*1 include the palindromic motifs. Similar palindromes are conserved in the ends of all group A IStrons (Figure 3). It should be noted that the subterminal palindromic sequences in the 5′ end would also be part of the stem of the P1 intron domain at the RNA level (Figure 4). The structure of the ends of IS*607* family transposons is different. The 5′ and 3′ ends contain complementary motifs and form imperfect AT-rich IRs (65). In this study we discovered that IS*Bth15*, an IS from *Bacillus thuringiensis subsp. kurstaki* YBT-1520, has a GG-ending target site and the 5′ and 3′ extremities matching those of the group B IStrons Cb*ISt*1 and Cb*ISt*4 (Figure 3), as well as sequence homology to those IStrons (51% amino-acid identity between the ORFs B). IS*Bth15* lacks ORF A and is thus classified as an IS*200*/IS*605* member solely on the basis of ORF B, however, as noted in ISfinder, it is highly similar to the IS*607* family elements IS*Cbt4* (which corresponds to Cb*ISt*3) and IS*Cbo6*, thus IS*Bth15* more likely belongs to the IS*607* class. While the sequence of the ends may vary among group B IStrons, they all include complementary AT-rich sequences (Figure 3).

In addition to homology between IStrons and IS elements, our searches also identified similarities between IStrons and two group IA2 introns from: (i) a number of *Fusobacterium* strains (*Fusobacterium periodonticum* ATCC 33693*, Fusobacterium sp.* 1_1_41FAA, 2_1_31 and F0437 oral taxon 370), the first three of which presumably encode IStrons (as suggested by the presence of unassembled partial IStron copies at the extremities of unfinished genomic scaffolds) and (ii) from two strains of the Firmicute *Turicibacter* (*T. sanguinis* PC909 and *Turicibacter sp.* HGF1). The *Fusobacterium* intron is closely related to the intron component of *Fusobacterium nucleatum* group A IStron Fn*ISt*1 and, like that one, is inserted next to a TTGAT motif (Figure 3). The *Turicibacter* intron is more similar to the group B IStrons Cb*ISt*1 and Cb*ISt*4 and, like those, has a GG-ending homing site (GGAGG). However, the two introns lack the IR stem-loop secondary structure, their 3′ ends do not match the 3′ termini of IStrons and both introns exhibit specific sequence motifs, all of this is indicating that they are distinct elements and not merely former group A or B IStron copies that have lost the entire IS component. The aforementioned group I introns and IS transposons may be related to the elements that were at the origin of IStrons.

To determine whether all IStrons have originated from a common ancestor, phylogenetic analyses of their ORFs were conducted. For each of the two IS families (IS*200*/IS*605* and IS*607*) and the two types of ORFs (ORF A and ORF B), the IStron ORFs were aligned to each other and with their closest homologues from IS elements, most of them being from bacterial species in which no IStron could be identified. As shown in Figure 5, there are IS element ORFs that are more closely related to IStron ORFs than IStrons ORFs are to each other. This is true for IS*200*/IS*605*-like ORFs A (group A IStrons) as well as for ORFs B (group A and B IStrons), which all belong to the IS*200*/IS*605* family. In these cases, IStrons do not form monophyletic clusters. In fact, in the ORF B tree IStrons from groups A and B are intermixed. Altogether, these results suggest that IStrons have multiple origins. Furthermore, there is a remarkable correlation between the ORF phylogenetic relationships and the IStron target site. That is, ORFs of IStrons that recognize the same target sequence are phylogenetically clustered (Figure 5). This indicates that IStrons (in different species) with the same target site derive from a common ancestor.

### Functional analysis of the Bc*ISt*1 IStron

To complement the genomic and bioinformatic data, exploratory experiments were conducted to test the functionality in terms of splicing and mobility of the group B IStron Bc*ISt*1 of *B. cereus* ATCC 10987.

### *In vivo* splicing of Bc*ISt*1 RNA

Like other group B IStrons, Bc*ISt*1 is particularly interesting because it exhibits an apparently unusual 5′ splice site where the 5′ exon does not end with a U. *B. cereus* ATCC 10987 harbors three copies of Bc*ISt*1 that are 96–99% identical to each other, all of them encoding a truncated ORF A and a full-length ORF B sequence (IStron length 1915–1916 bp).

The splicing activity was probed *in vivo* by RT-PCR experiments conducted on total RNA from *B. cereus* ATCC 10987. For each of the three Bc*ISt*1 copies RT-PCR with inward-oriented primers located in the exons generated the product of ∼300–400-bp (depending on the copy) corresponding to the spliced exons lacking the IStron sequence (Figure 6A). Inverse RT-PCR with outward-oriented primers located within the IStron generated products corresponding to the free, circularized, IStron in which the 3′ and 5′ ends are joined together (Figure 6B and C). These results demonstrate that the Bc*ISt*1 IStron element splices *in vivo* in *B. cereus*. Cloning and sequencing of the RT-PCR products validated the IStron boundaries determined previously by bioinformatic analysis. The U base following the AGGG target site was absent from the ligated exons’ sequence, but was present in one of the circular IStron junction products that thus represented the full-length IStron RNA circle (Figure 6B, black arrow, and Figure 6C). This confirmed that Bc*ISt*1 does splice after a G and not a U, and that the U base following the target site is indeed the first base of the IStron. In addition to the full-length IStron, sequences of the circular junction products revealed several partial circles in which the IStron lacked the first one, the first 42, the first 71 or the last base (Figure 6B, gray arrow, and Figure 6C). Altogether, the RT-PCR experiments indicate that the Bc*ISt*1 IStron has undergone the reactions of typical group I introns including the splicing, intramolecular cyclization (leading to shortened circles) and full-length circularization pathways.

### DNA reactions of Bc*ISt*1

In order to obtain a first insight into whether the Bc*ISt*1 IStron could be mobilized, a series of experiments were conducted in which the IStron and flanking exons were cloned into an expression vector transformed into *E. coli* (lacking IStrons; Figure 7A). The fate of the IStron construct was followed by Southern hybridization of DNA samples extracted from bacterial cultures during 8–12 days of growth, using labeled dsDNA probes specific for the intron or IS (ORF B) component of Bc*ISt*1 or the 5′ exon. A difference among the banding patterns obtained with the various probes would indicate that a DNA rearrangement (i.e. an excision event) involving the IStron has occurred. Linearized plasmid bands of interest were gel-purified and sequenced to characterize the rearrangement. During or at the end of these mobility experiments, the *E. coli* genomic DNA, purified of plasmid, was isolated to identify possible insertion events.

**Figure 8.**
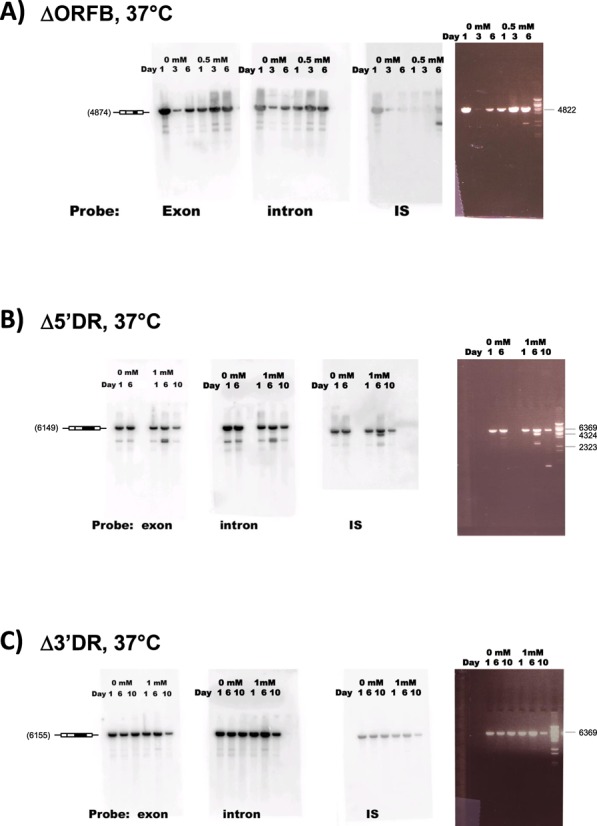
Test for Bc*ISt*1 IStron mobility using mutant Bc*ISt*1 constructs deleted of the sequence regions covering ORF B (panel **A**) or the 5′ DR and 3′ DR motifs (panels **B** and **C**, respectively). Data shown as explained in the legend to Figure 7. No IPTG, 0.5 mM or 1 mM IPTG was used. In all panels, plasmid DNA was linearized with *Nco*I. Note the absence of rearrangement events for the ΔORFB and Δ5′DR mutants, whereas events involving the IS component occurred with the Δ3′DR construct. DNA marker: Lambda-*BstE*II (New England Biolabs).

Besides growth of strains transformed with the WT IStron construct, similar growth experiments were conducted with constructs deleted of specific sequences, such as ORF B or IR motifs (Figure 7A). The fates of all constructs were compared to growth in genetically different recipient *E. coli* strains (RecA+ or RecA−) and at different temperatures (30, 37 or 42°C) to investigate the effects of host factors or growth conditions on possible mobility events, as observed for other mobile elements (70,71). Growth experiments were also conducted with or without IPTG to see if induction of the promoter upstream of the cloned IStron sequence could have an impact on Bc*ISt*1 mobility. The main findings are summarized below and are illustrated in Figures 7 and 8.

One of the most striking results was obtained with the WT IStron construct at 37°C without IPTG induction, in which case the loss of hybridization signal with the IS ORF B probe indicated that excision of the ORF (transposase) component out of Bc*ISt*1 occurred (Figure 7B, lower band corresponding to the ORF-less product). Remarkably, the boundaries of the excision point corresponded exactly to the sequence of the ORF-less Bc*ISt*1 copies encoded by some emetic *B. cereus* strains (Figure 4, boxed inset). That is, after ORF component excision only the IR motifs that would form the IR stem-loop structure in the IStron RNA remain in the IS region. Therefore, the experiment has reproduced an event that did occur during the natural evolution of *B. cereus*. Induction with IPTG or growth at lower (30°C) or higher (42°C) temperatures also led to ORF component excision, but with variable boundaries (data not shown). A common pattern, though, was that the termini of the excised regions were generally AT-rich, whereas the flanking sequences were usually GC-rich, a pattern reminiscent of the IStron ends. Under all growth conditions tested, sequencing of the plasmid inserts revealed no event corresponding to the full-length IStron excision. Importantly, no DNA rearrangement could be detected, under any condition, when the region covering the ORF B sequence was deleted from Bc*ISt*1, while keeping the IR motifs (ΔORFB construct; Figure 8A), suggesting that the IS excision events observed for the WT construct must be dependent on the activity of the ORF B protein. Furthermore, no clear event was detected with the WT construct when transformed into an *E. coli* strain lacking the RecA recombinase (Figure 7C), with or without induction by IPTG. This could suggest that excision of the ORF component of Bc*ISt*1 may require host factors, e.g. for recombination and/or repair. Moreover, no insertion events could be detected by hybridization of genomic DNA isolated during or after the growth experiments for any of the constructs.

Like other group B IStrons, the IS component of Bc*ISt*1 is flanked by a peculiar arrangement of direct and inverted repeats. That is, 15 nt (TAAATTTGATTGAAT) of the 21-nt 5′ IR motif in the IR stem are directly repeated on the 3′ side immediately upstream of the 3′ IR motif (Figure 3). The two copies of this direct repeat (DR) will be named hereafter 5′ DR and 3′ DR, respectively. The IR stem-loop of the ORF-less Bc*ISt*1 elements is made up of only one DR and the 3′ IR (Figure 4). This means that one of the DR motifs has been removed as part of the excision of the transposase component. We therefore tested the mobility of Bc*ISt*1 constructs deleted of either one of the 5′ DR and 3′ DR copies (Δ3′DR and Δ5′DR, respectively). Interestingly, no rearrangement event could be detected for the Δ3′DR construct, whereas a number of IS ORF excision events occurred for the Δ5′DR construct at 37°C with or without IPTG induction (Figure 8B and C). This suggests that the 3′ copy of the DR motif but not the 5′ copy may be required for ORF component excision, or at least that the 3′ DR is functionally more important than the 5′ DR. Interestingly, among the 66 full-length Bc*ISt*1 copies identified in various *B. cereus* group strains it appears that the 3′ DR is substantially more conserved than the 5′ DR (the 3′ DR is identical in 57 of the 66 Bc*ISt*1 sequences whereas more than half of the sequences show differences in the 5′ DR; data not shown). This conservation could be an additional argument supporting the functional relevance of the 3′ DR over the 5′ DR.

## DISCUSSION

In this study, we have conducted an extensive bioinformatic survey of chimeric IStron elements in sequenced genomes. Our analysis revealed that the two IStrons that were known so far, Cd*ISt*1 and Bc*ISt*1, are part of two major and diverse groups of IStrons encompassing 16 different elements (Table 1 and Figure 3). Intriguingly, IStrons were identified only in Bacteria of the Firmicutes and Fusobacteria phyla. Two of the search procedures employed in this study were designed to be generic and to identify IStrons on the basis of their structure and that may not be closely related at the sequence level (Figure 2), and even though some divergent IStrons may have been missed, this very restricted phylogenetic distribution is puzzling. Since IStrons have the capabilities of both self-splicing and transposition, one would expect IStrons to be powerful elements that should be able to spread and maintain themselves in genomes. So why are not IStrons more widespread? One hypothesis could be that they are evolutionarily recent elements that have not had time to proliferate. The fact that no separate copies of the intron and IS components of IStrons were found in any genome would on the contrary suggests that these components have been part of IStrons for a long time. While there is common ancestry between the group I intron parts of all IStrons (which belong to the IA2 structural class; Figure 4), the IS components and their ORFs are from different phylogenetic families and exhibit paraphyletic relationships (Figure 5), which strongly suggests that the symbiosis between group I introns and IS elements occurred multiple times in evolution. A possible scenario to explain the narrow distribution of IStrons may be that related group I introns of class IA2 have been transferred within and between Firmicutes and Fusobacteria, and then recently associated with different IS elements present in these species. Furthermore, to make a functional IStron requires the association of an intron and an IS that share similar target sites, and that must be present at the same time in a given organism, a low-probability situation that may have occurred only in a few bacteria such as Firmicutes and Fusobacteria. It is also possible that the physiological conditions in these organisms are most favorable or most suitable for IStron activity. Indeed, it has been shown that host identity and intracellular environment have an effect on mobile element function (70,71).

Like their phylogenetic distribution, the genomic distribution of IStrons is also of particular interest. IStron insertions are mostly intragenic and in protein-coding genes of diverse functions (Table 1), in contrast to group I introns which target conserved rRNA/tRNA genes and genes involved in DNA metabolism, and in contrast to IS elements which are mostly located outside coding regions. There is presumably no selection against intron or IStron insertion into genes since they are spliced out and would not necessarily affect gene expression. Nevertheless, one cannot rule out the possibility of polar downstream effects from transcription of the IS ORFs. Unlike HEG-encoding group I introns whose transposition is limited by the high site-specificity of their HEG, IStrons have less stringent sequence requirements as they target only a short 2–5 nt motif, which is present hundreds or thousands of times in the host genome, and thus IStrons can be present in multiple copies per strain (Table 1). The IStrons distribution reflects the activities of their two components: the lower stringency of IS transposases allows the invasion at multiple sites, including coding regions, and insertions are harmless due to the splicing ability conferred by the intron component. One would therefore expect IStrons to be randomly distributed in genomes and genes. Interestingly, however, more than half of intragenic IStron locations are in-frame within the host gene, as is the case for most group I introns that are located within protein-coding genes (see, e.g. (72,73)). Most IStrons, like many group I introns, start with the sequence UAA, UAG or UGA, and thus an in-frame insertion would create a stop codon at the insertion point. It has been shown for group I introns that such insertions prevent translating ribosomes from running into the intron structure, which would interfere with intron folding and splicing (74), and thus in-frame insertions are preferentially selected during evolution. Therefore, although the IS component provides a more promiscuous way of mobility than HEGs, it seems that the functional requirements for splicing somewhat limit the possibilities of insertion.

A striking and general feature of IStrons is the frequent degeneration of the IS part, especially in the ORF A region (Table 1). Whereas the mobility function may be lost, the splicing function appears to be always retained. In all cases tested, the splicing of Cd*ISt*1 IStron variants harboring truncated IS components was confirmed (39–41). Since IStrons are usually intragenic, maintenance of splicing function likely reflects a selection pressure to preserve host gene activity. Our survey suggests in addition that IStrons have been able to spread without ORF A, because IStrons sharing identical ORF A degenerations were found in multiple genomic locations. The possibility of mobilization *in trans* by an IStron copy encoding an intact ORF A seems unlikely because no such IStron is present in any of the genomes harboring truncated copies. Thus, only ORF B may be required for transposition after the IS has associated with the intron as part of an IStron, and IStrons are presumably mobile without ORF A but need ORF B. IStrons from group A and B encode an IS belonging to the IS*200*/IS*605* and IS*607* family, respectively. For the IS*200*/IS*605* family element IS*8301*/IS*Dra2* of *D. radiodurans*, which is related to Cd*ISt*1 (Figure 3), it has been shown that ORF A but not ORF B is essential for transposition (75) and that ORF B has a regulatory effect in inhibiting the ORF A-mediated excision and insertion steps of IS*8301*/IS*Dra2* transposition (76). Similarly, for IS*608* (from the IS*200*/IS*605* family) as well as IS*607* of *Helicobacter pylori* only ORF A is required for transposition, at least in *E. coli* (65–66,77). Nevertheless, and in contrast to group B IStrons, all but two of the 39 IS*607*-related members from diverse Bacteria and Archaea recorded in ISfinder do encode two complete, intact ORFs. To explain this, it has been suggested that either ORF may be important for IS*607* mobility in different organisms or that ORF B may have a regulatory role or another function independent of transposition (65). In any case, the situation in IStrons, where ORF A rather than ORF B appears to be dispensable, is different from that in IS*200*/IS*605* and IS*607* transposons and warrants functional study, and is probably linked to the establishment of the intron–IS symbosis.

In the course of this study, we made the first attempt at testing the mobility of an IStron under various experimental conditions using the Bc*ISt*1 element from *B. cereus* and *E. coli* as a host. Whereas evidence for IStron mobility could not be obtained, various DNA rearrangements involving the partial or full excision of the region containing the ORF (transposase) were detected (Figure 7B and C). In particular, excision of the entire ORF component occurred, generating an ORF-less IStron with boundaries identical to those of the ORF-less elements identified in the genomes of emetic *B. cereus* strains. Since the rearrangements were observed only when using IStron constructs encoding a full-length ORF B sequence, one can presume that they are dependent on the action of ORF B (Figure 8A). The observed excisions of the IS component may be by-products of ORF B activity. Another possibility is that excision occurs via replication slippage due to the presence of DR and IR motifs flanking the ORF region, as suggested for other transposons (70,78), which might explain the differential effects of the 5′ DR and 3′ DR repeats on ORF component excision (Figure 8B and C). The excision event that occurred in emetic *B. cereus* strains maintains the IStron secondary structure in the IR region (Figure 4), explaining why it has been retained. The fact that mobility of the full-length IStron as one unit was not detected could be because excision of the ORF region may be more likely than (or is favored over) IStron excision in the *E. coli* heterologous host. IStron mobility may have more stringent requirements on *B. cereus*-specific host factors, although no mobility or excision event was detected when a *B. cereus*-specific plasmid carrying the Bc*ISt*1 WT construct was transfected and grown in the *B. cereus* type strain ATCC 14579 that does not harbor any copies of Bc*ISt*1 (data not shown). It could also be that the IStron does not actually excise and does not move via a ‘cut-and-paste’ transposition mechanism, but rather transposes via a ‘copy-and-paste’ mechanism in which the donor copy remains in its original genomic location (12). However, in a ‘copy-and-paste’ mechanism one would expect many copies to be shared among related strains. IStron insertion loci are mostly specific to each strain, which would rather suggest a ‘cut-and-paste’ mechanism involving IStron excision. Sequence features that have been demonstrated or presumed to be important for recognition of transposon ends by the transposases, such as palindromic motifs in IS*200*/IS*605* elements (69) and imperfect AT-rich IRs in IS*607* elements (65), are present in group A and B IStrons, respectively (Figures 3 and 4). From that, one could assume that IStron mobility occurs via the same mechanisms as those employed by the IS*200*/IS*605* and IS*607* transposons. Both types of transposons move by an ORF A-mediated mechanism involving excision of a circular intermediate (63,67–69). However, the frequent degeneration of ORF A in IStrons would imply that there are differences. A lot remains to be learned and understood about IStron mobility and to decipher the transposition mechanism and the actual functions of ORF A and ORF B in both groups of IStrons.

We also probed the splicing activity of Bc*ISt*1. While splicing of Cd*ISt*1, a group A IStron has been shown previously by Braun *et al.* (39,40), our study provides the first analysis of a group B IStron. We here have presented experimental evidence that Bc*ISt*1 splices after a G instead of a U, unlike for virtually all known group I introns and group A IStrons (Figures 3 and 6; (31,32)). These results warrant more functional analyses of the 5′ splice site selection by the ribozyme of group B IStrons. A question to answer would be whether the one-base shift in 5′ splice site is related to the position of the critical U–G pair within the P1 stem structure, in which it is predicted to be flanked by a loop instead of by one or more base-pairs as is the case for most group I introns and group A IStrons (Figure 4).

IStrons are ‘chimeras of chimeras’ because the IS*200*/IS*605* and IS*607* elements that have associated with the group I intron to form a chimeric IStron are themselves chimeric as they encode ORFs A and B that are of different phylogenetic origins (Figure 5; (6,60,65)). IStrons give therefore an additional example of the modular evolution of mobile elements. Indeed, many transposable elements from prokaryotes and eukaryotes are composed of ORFs from different origins that carry similar (e.g. transposase) or distinct functions (6,10,18), and in some cases genetic elements of different kinds have associated to form composite elements, involving self-splicing introns in particular. In addition to IStrons, HEG-containing group I introns were made by the association of a self-splicing ribozyme with a mobile HEG gene, and reverse-transcriptase (RT)-containing group II introns were created by the association between a self-splicing ribozyme and an RT gene (79). In these cases, the relationship between the elements can be considered symbiotic because the elements carry out functions that are beneficial to each other: the IS, HEG or RT promotes mobility while the ribozyme allows splicing and thus avoids damage to the host. A fundamental question is how did these chimeric elements actually form? In the case of HEG-containing introns two likely scenarios have been proposed (23). Both have been derived from work on phages. In one scenario, the formation of an HEG-containing intron happened due to the fortuitous occurrence within the intron of a sequence matching the HEG target site (80). As the HEG makes a double-stranded break at the target site, it was then copied into the intron during the DNA repair process. In the other scenario, called ‘collaborative homing’, the merging of intron and HEG occurred when the HEG target site happened to match the intron insertion site (81,82). An illegitimate recombination event is invoked to insert the HEG within the ribozyme structure. IS elements of the IS*200*/IS*605* and IS*607* families are site-specific and therefore both scenarios could be similarly envisioned to explain the formation of IStrons. Even though there is no recognizable sequence similarity between the regions flanking the IStron and those flanking the IS component, the similarity may have been lost during evolution. The finding of IS elements and introns exhibiting termini and/or target sites corresponding to those of IStrons could support the second scenario (Figure 3; (39,41)). During evolution HEG-containing group I introns have been invaded independently by HEGs which have been inserted in various terminal loops of the intron structure (in P1, P2, P5, P6, P8 or P9 domains), a variability that would reflect illegitimate recombination. As explained above, the association between group I introns and IS elements seems to have also been established independently several times. Remarkably, however, the relative location of the IS component is the same in all IStrons (i.e. at the 3′ end of the intron downstream of the P9.2 subdomain; Figure 4), and, reminiscent of HEG-containing introns, it is also within a terminal stem-loop region at the periphery of the intron structure. From this fixed structural organization IStrons seem to be the result of the fusion of IS elements at the 3′ end of group I ribozymes (39,41). The homology between the 5′ and 3′ termini of IS elements and IStrons (Figure 3) could lead to the hypothesis that IStrons have been created by homologous recombination between IS and introns sharing similar ends. Or could it be homing of introns into IS elements? Whatever mechanism, it was such that the merging event always placed the IS component at the same location, and thus was probably different from the process that has led to the formation of HEG-containing group I introns. Additional sequence and structure/function analyses are necessary to better understand how IStrons function and thus provide insights into the process that has led to the formation of these chimeric genetic elements.

## SUPPLEMENTARY DATA

Supplementary Data are available at NAR Online.

SUPPLEMENTARY DATA

## References

[1] Frost L.S., Leplae R., Summers A.O., Toussaint A. (2005). Mobile genetic elements: the agents of open source evolution. Nat. Rev..

[2] Aziz R.K., Breitbart M., Edwards R.A. (2010). Transposases are the most abundant, most ubiquitous genes in nature. Nucleic Acids Res..

[3] Hazen T.H., Pan L., Gu J.D., Sobecky P.A. (2010). The contribution of mobile genetic elements to the evolution and ecology of Vibrios. FEMS Microbiol. Ecol..

[4] Kazazian H.H. (2004). Mobile elements: drivers of genome evolution. Science.

[5] Lisch D. (2013). How important are transposons for plant evolution. Nat. Rev. Genet..

[6] Chandler M., Mahillon J., Craig N.L., Craigie R., Gellert M., Lambowitz A.M. (2002). Insertion sequences revisited. Mobile DNA II.

[7] Feschotte C., Pritham E.J. (2007). DNA transposons and the evolution of eukaryotic genomes. Annu. Rev. Genet..

[8] Mahillon J., Chandler M. (1998). Insertion sequences. Microbiol. Mol. Biol. Rev..

[9] Siguier P., Filée J., Chandler M. (2006). Insertion sequences in prokaryotic genomes. Curr. Opin. Microbiol..

[10] Wicker T., Sabot F., Hua-Van A., Bennetzen J.L., Capy P., Chalhoub B., Flavell A., Leroy P., Morgante M., Panaud O. (2007). A unified classification system for eukaryotic transposable elements. Nat. Rev. Genet..

[11] Siguier P., Gourbeyre E., Chandler M. (2014). Bacterial insertion sequences: their genomic impact and diversity. FEMS Microbiol. Rev..

[12] Curcio M.J., Derbyshire K.M. (2003). The outs and ins of transposition: from mu to kangaroo. Nat. Rev. Mol. Cell Biol..

[13] Hickman A.B., Chandler M., Dyda F. (2010). Integrating prokaryotes and eukaryotes: DNA transposases in light of structure. Crit. Rev. Biochem. Mol. Biol..

[14] Kapitonov V.V., Jurka J. (2007). Helitrons on a roll: eukaryotic rolling-circle transposons. Trends Genet..

[15] Toleman M.A., Bennett P.M., Walsh T.R. (2006). ISCR elements: novel gene-capturing systems of the 21st century. Microbiol. Mol. Biol. Rev..

[16] Filée J., Siguier P., Chandler M. (2007). Insertion sequence diversity in archaea. Microbiol. Mol. Biol. Rev..

[17] Siguier P., Perochon J., Lestrade L., Mahillon J., Chandler M. (2006). ISfinder: the reference centre for bacterial insertion sequences. Nucleic Acids Res..

[18] Kapitonov V.V., Jurka J. (2008). A universal classification of eukaryotic transposable elements implemented in Repbase. Nat. Rev. Genet..

[19] Craig N.L. (1997). Target site selection in transposition. Annu. Rev. Biochem..

[20] Belfort M., Derbyshire V., Parker M.M., Cousineau B., Lambowitz A.M., Craig N.L., Craigie R., Gellert M., Lambowitz A.M. (2002). Mobile introns: pathways and proteins. Mobile DNA II.

[21] Cech T.R. (1990). Self-splicing of group I introns. Annu. Rev. Biochem..

[22] Haugen P., Simon D.M., Bhattacharya D. (2005). The natural history of group I introns. Trends Genet..

[23] Hausner G., Hafez M., Edgell D.R. (2014). Bacterial group I introns: mobile RNA catalysts. Mobile DNA.

[24] Hougland J.L., Piccirilli J.A., Forconi M., Lee J., Herschlag D., Gesteland R.F., Cech T.R., Atkins J.F. (2006). How the group I intron works: a case study of RNA structure and function. The RNA World.

[25] Michel F., Westhof E. (1990). Modelling of the three-dimensional architecture of group I catalytic introns based on comparative sequence analysis. J. Mol. Biol..

[26] Stahley M.R., Strobel S.A. (2006). RNA splicing: group I intron crystal structures reveal the basis of splice site selection and metal ion catalysis. Curr. Opin. Struct. Biol..

[27] Vicens Q., Cech T.R. (2006). Atomic level architecture of group I introns revealed. Trends Biochem. Sci..

[28] Woodson S.A. (2005). Structure and assembly of group I introns. Curr. Opin. Struct. Biol..

[29] Edgell D.R., Belfort M., Shub D.A. (2000). Barriers to intron promiscuity in bacteria. J. Bacteriol..

[30] Gardner P.P., Daub J., Tate J.G., Nawrocki E.P., Kolbe D.L., Lindgreen S., Wilkinson A.C., Finn R.D., Griffiths-Jones S., Eddy S.R. (2009). Rfam: updates to the RNA families database. Nucleic Acids Res..

[31] Zhou Y., Lu C., Wu Q.J., Wang Y., Sun Z.T., Deng J.C., Zhang Y. (2008). GISSD: Group I intron sequence and structure database. Nucleic Acids Res..

[32] Cannone J.J., Subramanian S., Schnare M.N., Collett J.R., D'Souza L.M., Du Y., Feng B., Lin N., Madabusi L.V., Muller K.M. (2002). The comparative RNA web (CRW) site: an online database of comparative sequence and structure information for ribosomal, intron, and other RNAs. BMC Bioinformatics.

[33] Stoddard B.L. (2005). Homing endonuclease structure and function. Q. Rev. Biophys..

[34] Stoddard B.L. (2014). Homing endonucleases from mobile group I introns: discovery to genome engineering. Mobile DNA.

[35] Lang B.F., Laforest M.J., Burger G. (2007). Mitochondrial introns: a critical view. Trends Genet..

[36] Meng Q., Zhang Y., Liu X.Q. (2007). Rare group I intron with insertion sequence element in a bacterial ribonucleotide reductase gene. J. Bacteriol..

[37] Nielsen H., Johansen S.D. (2009). Group I introns: Moving in new directions. RNA Biol..

[38] Nielsen H., Westhof E., Johansen S. (2005). An mRNA is capped by a 2′, 5′ lariat catalyzed by a group I-like ribozyme. Science.

[39] Braun V., Mehlig M., Moos M., Rupnik M., Kalt B., Mahony D.E., von Eichel-Streiber C. (2000). A chimeric ribozyme in *Clostridium difficile* combines features of group I introns and insertion elements. Mol. Microbiol..

[40] Hasselmayer O., Braun V., Nitsche C., Moos M., Rupnik M., von Eichel-Streiber C. (2004). *Clostridium difficile* IStron Cd*ISt1*: discovery of a variant encoding two complete transposase-like proteins. J. Bacteriol..

[41] Hasselmayer O., Nitsche C., Braun V., von Eichel-Streiber C. (2004). The IStron Cd*ISt*1 of *Clostridium difficile*: molecular symbiosis of a group I intron and an insertion element. Anaerobe.

[42] Tourasse N.J., Helgason E., Økstad O.A., Hegna I.K., Kolstø A.B. (2006). The *Bacillus cereus* group: novel aspects of population structure and genome dynamics. J. Appl. Microbiol..

[43] Benson D.A., Karsch-Mizrachi I., Clark K., Lipman D.J., Ostell J., Sayers E.W. (2012). GenBank. Nucleic Acids Res..

[44] Altschul S.F., Madden T.L., Schaffer A.A., Zhang J., Zhang Z., Miller W., Lipman D.J. (1997). Gapped BLAST and PSI-BLAST: a new generation of protein database search programs. Nucleic Acids Res..

[45] Nawrocki E.P., Kolbe D.L., Eddy S.R. (2009). Infernal 1.0: inference of RNA alignments. Bioinformatics.

[46] Gautheret D., Lambert A. (2001). Direct RNA motif definition and identification from multiple sequence alignments using secondary structure profiles. J. Mol. Biol..

[47] Gardner P.P., Daub J., Tate J., Moore B.L., Osuch I.H., Griffiths-Jones S., Finn R.D., Nawrocki E.P., Kolbe D.L., Eddy S.R. (2011). Rfam: Wikipedia, clans and the ‘decimal’ release. Nucleic Acids Res..

[48] Siguier P., Varani A., Perochon J., Chandler M. (2012). Exploring bacterial insertion sequences with ISfinder: objectives, uses, and future developments. Methods Mol. Biol..

[49] Zuker M. (2003). Mfold web server for nucleic acid folding and hybridization prediction. Nucleic Acids Res..

[50] Larkin M.A., Blackshields G., Brown N.P., Chenna R., McGettigan P.A., McWilliam H., Valentin F., Wallace I.M., Wilm A., Lopez R. (2007). Clustal W and Clustal X version 2.0. Bioinformatics.

[51] Dang C.C., Lefort V., Le V.S., Le Q.S., Gascuel O. (2011). ReplacementMatrix: a web server for maximum-likelihood estimation of amino acid replacement rate matrices. Bioinformatics.

[52] Anisimova M., Gil M., Dufayard J.F., Dessimoz C., Gascuel O. (2011). Survey of branch support methods demonstrates accuracy, power, and robustness of fast likelihood-based approximation schemes. Syst. Biol..

[53] Guindon S., Dufayard J.F., Lefort V., Anisimova M., Hordijk W., Gascuel O. (2010). New algorithms and methods to estimate maximum-likelihood phylogenies: assessing the performance of PhyML 3.0. Syst. Biol..

[54] Tourasse N.J., Stabell F.B., Reiter L., Kolstø A.B. (2005). Unusual group II introns in bacteria of the *Bacillus cereus* group. J. Bacteriol..

[55] Stabell F.B., Tourasse N.J., Ravnum S., Kolstø A.B. (2007). Group II intron in *Bacillus cereus* has an unusual 3′ extension and splices 56 nucleotides downstream of the predicted site. Nucleic Acids Res..

[56] Reiter L., Tourasse N.J., Fouet A., Loll R., Davison S., Okstad O.A., Piehler A.P., Kolsto A.B. (2011). Evolutionary history and functional characterization of three large genes involved in sporulation in Bacillus cereus group bacteria. J. Bacteriol..

[57] Tourasse N.J., Økstad O.A., Kolstø A.B. (2010). HyperCAT: an extension of the SuperCAT database for global multi-scheme and multi-datatype phylogenetic analysis of the Bacillus cereus group population. Database (Oxford).

[58] Kolstø A.B., Tourasse N.J., Økstad O.A. (2009). What sets *Bacillus anthracis* apart from other *Bacillus* species?. Annu. Rev. Microbiol..

[59] Rasko D.A., Altherr M.R., Han C.S., Ravel J. (2005). Genomics of the *Bacillus cereus* group of organisms. FEMS Microbiol. Rev..

[60] Kersulyte D., Akopyants N.S., Clifton S.W., Roe B.A., Berg D.E. (1998). Novel sequence organization and insertion specificity of IS605 and IS606: chimaeric transposable elements of Helicobacter pylori. Gene.

[61] Chandler M., de la Cruz F., Dyda F., Hickman A.B., Moncalian G., Ton-Hoang B. (2013). Breaking and joining single-stranded DNA: the HUH endonuclease superfamily. Nat. Rev..

[62] Ronning D.R., Guynet C., Ton-Hoang B., Perez Z.N., Ghirlando R., Chandler M., Dyda F. (2005). Active site sharing and subterminal hairpin recognition in a new class of DNA transposases. Mol. Cell.

[63] Boocock M.R., Rice P.A. (2013). A proposed mechanism for IS607-family serine transposases. Mobile DNA.

[64] Smith M.C., Thorpe H.M. (2002). Diversity in the serine recombinases. Mol. Microbiol..

[65] Kersulyte D., Mukhopadhyay A.K., Shirai M., Nakazawa T., Berg D.E. (2000). Functional organization and insertion specificity of IS*607*, a chimeric element of *Helicobacter pylori*. J. Bacteriol..

[66] Kersulyte D., Velapatino B., Dailide G., Mukhopadhyay A.K., Ito Y., Cahuayme L., Parkinson A.J., Gilman R.H., Berg D.E. (2002). Transposable element IS*Hp608* of *Helicobacter pylori*: nonrandom geographic distribution, functional organization, and insertion specificity. J. Bacteriol..

[67] Guynet C., Hickman A.B., Barabas O., Dyda F., Chandler M., Ton-Hoang B. (2008). In vitro reconstitution of a single-stranded transposition mechanism of IS608. Mol. Cell.

[68] He S., Guynet C., Siguier P., Hickman A.B., Dyda F., Chandler M., Ton-Hoang B. (2013). IS200/IS605 family single-strand transposition: mechanism of IS608 strand transfer. Nucleic Acids Res..

[69] He S., Hickman A.B., Dyda F., Johnson N.P., Chandler M., Ton-Hoang B. (2011). Reconstitution of a functional IS608 single-strand transpososome: role of non-canonical base pairing. Nucleic Acids Res..

[70] Nagy Z., Chandler M. (2004). Regulation of transposition in bacteria. Res. Microbiol..

[71] Coros C.J., Landthaler M., Piazza C.L., Beauregard A., Esposito D., Perutka J., Lambowitz A.M., Belfort M. (2005). Retrotransposition strategies of the *Lactococcus lactis* Ll.LtrB group II intron are dictated by host identity and cellular environment. Mol. Microbiol..

[72] Lazarevic V. (2001). Ribonucleotide reductase genes of *Bacillus* prophages: a refuge to introns and intein coding sequences. Nucleic Acids Res..

[73] Tourasse N.J., Kolstø A.B. (2008). Survey of group I and group II introns in 29 sequenced genomes of the *Bacillus cereus* group: insights into their spread and evolution. Nucleic Acids Res..

[74] Öhman-Hedén M., Åhgren-Stålhandske A., Hahne S., Sjöberg B.M. (1993). Translation across the 5′-splice site interferes with autocatalytic splicing. Mol. Microbiol..

[75] Pasternak C., Ton-Hoang B., Coste G., Bailone A., Chandler M., Sommer S. (2010). Irradiation-induced *Deinococcus radiodurans* genome fragmentation triggers transposition of a single resident insertion sequence. PLoS Genet..

[76] Pasternak C., Dulermo R., Ton-Hoang B., Debuchy R., Siguier P., Coste G., Chandler M., Sommer S. (2013). IS*Dra2* transposition in *Deinococcus radiodurans* is downregulated by TnpB. Mol. Microbiol..

[77] Ton-Hoang B., Guynet C., Ronning D.R., Cointin-Marty B., Dyda F., Chandler M. (2005). Transposition of ISHp608, member of an unusual family of bacterial insertion sequences. EMBO J..

[78] Bzymek M., Lovett S.T. (2001). Instability of repetitive DNA sequences: the role of replication in multiple mechanisms. Proc. Natl. Acad. Sci. U.S.A..

[79] Edgell D.R., Chalamcharla V.R., Belfort M. (2011). Learning to live together: mutualism between self-splicing introns and their hosts. BMC Biol..

[80] Loizos N., Tillier E.R., Belfort M. (1994). Evolution of mobile group I introns: recognition of intron sequences by an intron-encoded endonuclease. Proc. Natl. Acad. Sci. U.S.A..

[81] Bonocora R.P., Shub D.A. (2009). A likely pathway for formation of mobile group I introns. Curr. Biol..

[82] Zeng Q., Bonocora R.P., Shub D.A. (2009). A free-standing homing endonuclease targets an intron insertion site in the psbA gene of cyanophages. Curr. Biol..

