# Supplementary material for: Survey of chimeric IStron elements in bacterial genomes: multiple molecular symbioses between group I intron ribozymes and DNA transposons
Source: Nucleic Acids Res. 2014 Oct 16;42(20):12333–51. doi: 10.1093/nar/gku939 (PMC4227781; doi:10.1093/nar/gku939)
Supplement: SUPPLEMENTARY DATA [file supp_42_20_12333__index.html]

Survey of chimeric IStron elements in bacterial genomes: multiple molecular symbioses between group I intron ribozymes and DNA transposons — Survey of chimeric IStron elements in bacterial genomes: multiple molecular symbioses between group I intron ribozymes and DNA transposons — SUPPLEMENTARY DATA 

# Survey of chimeric IStron elements in bacterial genomes: multiple molecular symbioses between group I intron ribozymes and DNA transposons

## SUPPLEMENTARY DATA

**Files in this Data Supplement:**

- SUPPLEMENTARY DATA
