# Supplementary material for: Survey of chimeric IStron elements in bacterial genomes: multiple molecular symbioses between group I intron ribozymes and DNA transposons
Source: Nucleic Acids Res. 2014 Oct 16;42(20):12333–51. doi: 10.1093/nar/gku939 (PMC4227781; doi:10.1093/nar/gku939)
Supplement: SUPPLEMENTARY DATA [file supp_gku939_nar-01054-survey-d-2014-File010.zip › IStron_paper_supptable2_v2.doc]

**Supplementary Table S2**. Primers used in the study.

| RT-PCR primers pairs | |
| --- | --- |
| BcISt1a**^#^** right | ATT GCC GAA GTG AAC CAC AT |
| BcISt1a**^#^** left | GGG CAG AGA TTG TCA CGA AA |
| BcISt1b**^#^** left | CAT TGG GAT AGA CCG GAA GA |
| BcISt1b**^#^** right | TGA TGC CAC TGT CCC TAA AA |
| BcISt1c**^#^** left | GCG CTG GAA GTT TCT TAT GC |
| BcISt1c**^#^** right | CAC TGA GCC CGT TTT TGT TT |
| IStron circular junction left | CTG CCT GCT GTG GAG TGT TA |
| IStron circular junction right | TTT CCA CCA TAG GCT TG |
| Deletion construct primers | |
| ΔORFB right | GTT CAG GCG TTG GAA TCAGT |
| Δ3’DR left | TGT ATA GAT TTA GTT AAG TTT ATC GTA TCG |
| Δ3’DR right | CAC GAA GTT AGT GCT TCA TCG TGT T |
| Δ5’DR left | CTA TCA ACT TCA TTG ATT TTA TCG AA |
| Δ5’DR right | TCT TGT TCA ACT AAG GGC GC |
| PCR primers for amplification of hybridization probes | |
| 5’exon left | GCG CTG GAA GTT TCT TAT GC |
| 5’exon right | CAC GAA GAA TCG TTT GTG TCA |
| ORFB left | TAG AAA CCA TGC TGG TGC TG |
| ORFB right | CGC CGC ATC AAA ATC TCT |
| IStron probe left | GGG CAA TCC CTA AAG CTG AT |
| IStron probe right | TTT CCA CCA CCA TAG GCT TG |

**^#^**Bc*ISt*1a, b, and c correspond to the three IStron copies in *B. cereus* ATCC 10987 located at chromosomal positions 1109893–1107979, 2995638–2993724, and 3235386–3233471, respectively (Tourasse, Helgason, *et al.* 2006, J Appl Microbiol **101**(3): 579-593).
